# Supplementary material for: Novel Driver Strength Index highlights important cancer genes in TCGA PanCanAtlas patients
Source: PeerJ. 2022 Aug 11;10:e13860. doi: 10.7717/peerj.13860 (PMC9375969; doi:10.7717/peerj.13860)
Supplement: Supplemental Information 2 [file peerj-10-13860-s002.zip › COHORTS/genes plots/detailed/2021_8_16_14_9_distribution_events_detailed_27.pdf]

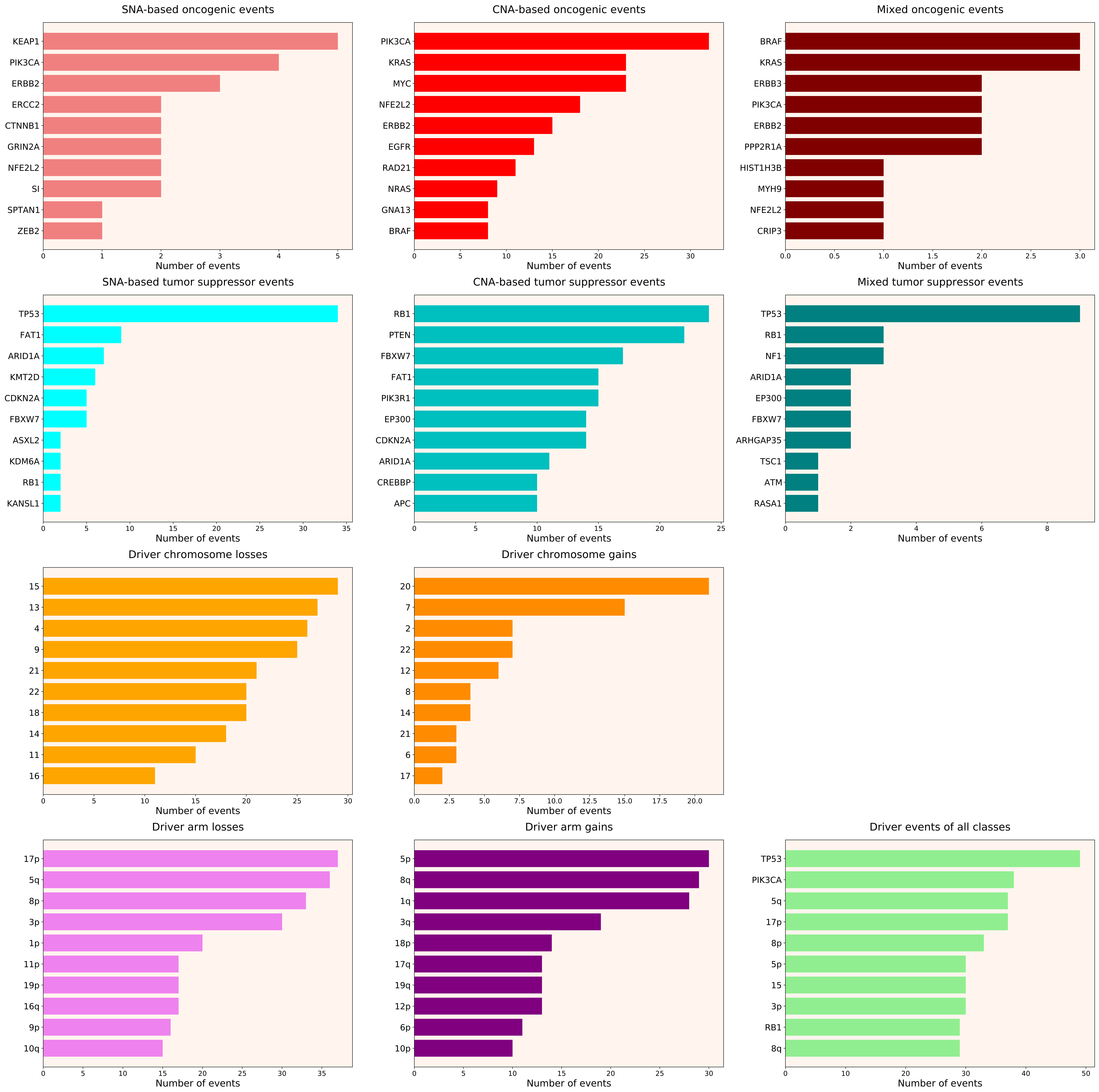

SNA-based oncogenic events
KEAP1
PIK3CA
ERBB2
ERCC2
CTNNB1
GRIN2A
NFE2L2
SI
SPTAN1
ZEB2
Number of events
CNA-based oncogenic events
PIK3CA
KRAS
MYC
NFE2L2
ERBB2
EGFR
RAD21
NRAS
GNA13
BRAF
Number of events
Mixed oncogenic events
BRAF
KRAS
ERBB3
PIK3CA
ERBB2
PPP2R1A
HIST1H3B
MYH9
NFE2L2
CRIP3
Number of events
SNA-based tumor suppressor events
TP53
FAT1
ARID1A
KMT2D
CDKN2A
FBXW7
ASXL2
KDM6A
RB1
KANSL1
Number of events
CNA-based tumor suppressor events
RB1
PTEN
FBXW7
FAT1
PIK3R1
EP300
CDKN2A
ARID1A
CREBBP
APC
Number of events
Mixed tumor suppressor events
TP53
RB1
NF1
ARID1A
EP300
FBXW7
ARHGAP35
TSC1
ATM
RASA1
Number of events
Driver chromosome losses
15
13
4
9
21
22
18
14
11
16
Number of events
Driver chromosome gains
20
7
2
22
12
8
14
21
6
17
Number of events
Driver arm losses
17p
5q
8p
3p
1p
11p
19p
16q
9p
10q
Number of events
Driver arm gains
5p
8q
1q
3q
18p
17q
19q
12p
6p
10p
Number of events
Driver events of all classes
TP53
PIK3CA
5q
17p
8p
5p
15
3p
RB1
8q
Number of events
